# Immune checkpoint inhibition in *Epstein-Barr virus* associated gastric cancer: a case report highlighting the role of biomarker-driven treatment selection

**DOI:** 10.3389/fonc.2026.1772402

**Published:** 2026-04-13

**Authors:** Jesús Yaringaño, Andrea Blázquez-López, Daniel Acosta, Stefania Landolfi, Sandra Castro-Boix, Susana Aguilar, Ana Vivancos, Elena Élez, Eduardo Terán-Brage

**Affiliations:** 1 Medical Oncology Department, Vall d’Hebron University Hospital, Barcelona, Spain; 2 Digestive Tumors Unit, Vall d’Hebron University Hospital, Barcelona, Spain; 3 Pathology Department, Vall d’Hebron University Hospital, Barcelona, Spain; 4 Esophagogastric Surgery Unit, General and Digestive Surgery Department, Vall d’Hebron University Hospital, Barcelona, Spain; 5 Molecular Prescreening Program, Vall d’Hebron Institute of Oncology, Barcelona, Spain; 6 Cancer Genomics Lab, Vall d’Hebron Institute of Oncology, Barcelona, Spain

**Keywords:** biomarker overlap, EBV - Epstein-Barr virus, gastric cancer, gastroesophageal adenocarcinoma, immune checkpoint inhibitor, nivolumab

## Abstract

Gastroesophageal adenocarcinoma (GEA) represents a heterogenous disease with poor prognosis, in which biomarker-driven strategies have gained relevance to optimize treatment selection. Epstein-Barr virus positive GEA (EBV-GEA) is an immune-enriched molecular subtype defined by TCGA suggested to have potential sensitivity to immune checkpoint inhibitors. However, clinical evidence supporting the efficacy of immunotherapy in this subgroup remains limited, and EBV testing is not yet considered a validated biomarker in international clinical guidelines. We report a case of a 74-year-old male with locally advanced, HER-2 negative, mismatch repair-proficient, EBV-positive gastric adenocarcinoma, with high PD-L1 expression (CPS 69), who experienced disease progression during perioperative FLOT chemotherapy. The patient subsequently received first-line treatment with nivolumab plus FOLFOX, achieving a marked radiological response. Salvage surgery revealed a complete pathological response and no further treatment with nivolumab was administered after surgery. This case illustrates the potential benefit of immunotherapy in EBV-positive gastric cancer, even in the microsatellite-stable setting. The overlap of biomarkers in this patient may have contributed to immune response, highlighting the importance of comprehensive molecular profiling in GEA and supporting EBV status as a promising predictor of immunotherapy benefit.

## Introduction

Gastroesophageal adenocarcinoma (GEA) represents the fifth most commonly diagnosed cancer worldwide, and the fifth leading cause of cancer-related death (1). Despite its high incidence and recent advances in systemic therapies, the prognosis of advanced GEA remains poor. Chemotherapy doublet constitutes the cornerstone of treatment, yielding a median overall survival (OS) of around 12 months in pivotal trials (2). The optimal use of predictive biomarkers to inform personalized therapy in GEA continues to be an unmet clinical need. Current ESMO guidelines recommend biomarker testing of microsatellite instability (MSI)/mismatch repair (MMR) proteins, human epidermal growth factor receptor 2 (HER2), programmed death-ligand 1 (PD-L1) expression and Claudin 18.2 (CLDN18.2) in advanced GEA to guide first-line treatment decisions in advanced disease (3–6).

Several phase III trials and subgroup analyses have demonstrated survival benefit from adding anti- programmed cell death-1 (anti-PD-1) monoclonal antibodies to chemotherapy in patients with MSI-high/deficient MMR or PD-L1 positive tumors. For this indication, pembrolizumab, nivolumab and tislelizumab have been approved by the European Medicines Agency. These findings underscore the critical role of biomarker-driven patient selection for immunotherapy in GEA (7–10).

Other biomarkers, not yet validated and, therefore, not widely used as routine practice, are tumor mutational burden (TMB) and Epstein-Barr Virus (EBV) status.

The Cancer Genome Atlas (TCGA) classification of GEA, proposed in 2014, has further advanced our understanding of the disease’s molecular heterogeneity and has enabled its stratification into four different molecular subtypes (11). Epstein-Barr virus-positive GEA (EBV-GEA), one of the defined subgroups, accounts for approximately 9% of cases and is characterized by unique molecular and immunological features. These include frequent *PIK3CA* mutations, DNA hypermethylation, *JAK2* amplification, and marked overexpression of PD-L1/PD-L2, along with a highly infiltrated immune microenvironment (12, 13). Notably, advanced/metastatic EBV-GEA exhibits an immune-enriched phenotype, with increased cytotoxic T-cell infiltration and enrichment of immune-related gene expression signatures (14), despite typically displaying a low TMB.

These immunological characteristics suggest that EBV-GEA patients may be suitable candidates for an immunotherapy-based approach. However, clinical evidence supporting the efficacy of immune checkpoint inhibitors (ICI) in this subset remains limited. We report a case of locally advanced gastric adenocarcinoma (ADC) characterized by pMMR status and EBV and PD-L1 positivity. Despite disease progression during perioperative FLOT, nivolumab treatment induced sufficient tumor regression to enable salvage surgery after 10 cycles, achieving a complete pathological response.

## Case description

We present a 74-year-old male with no known drug allergies, former smoker (53 pack-years), medical history of arterial hypertension and dyslipidemia, chronic hepatitis B virus infection under on antiviral prophylaxis, and prior uncomplicated duodenal ulcer. He was diagnosed with prostate adenocarcinoma in 2007, treated with radical prostatectomy and adjuvant radiotherapy.

**Figure 1:**
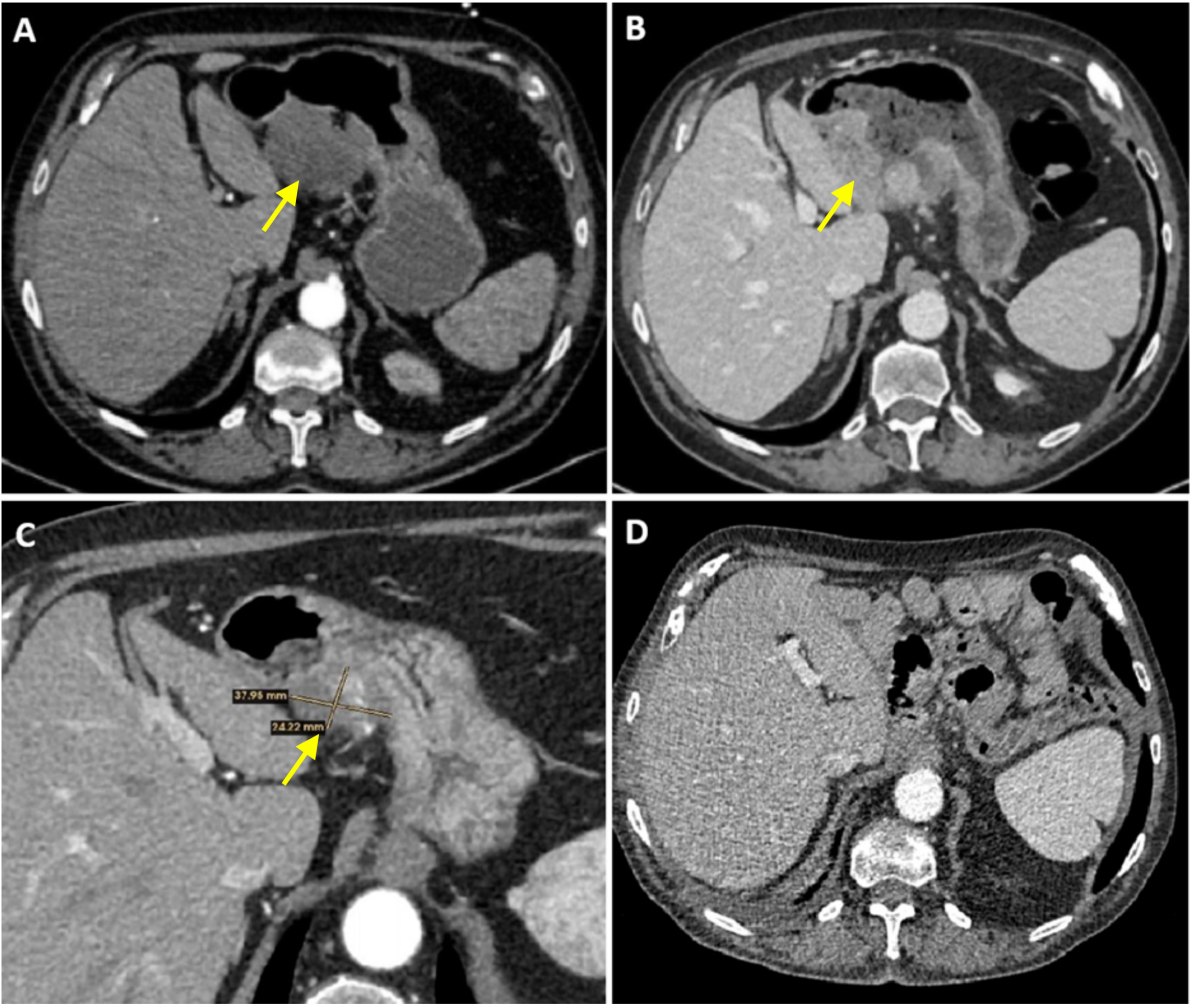
**(A)** Abdominal CT scan at diagnosis, describing a prominent exophytic mural lesion on the mesenteric border of the gastric antrum. **(B)** Abdominal CT scan in September 2024 revealing a loco-regional progression despite 4 cycles of FLOT chemotherapy. **(C)** Abdominal CT scan in December 2024 shows a partial tumor response after seven cycles of nivolumab monotherapy. **(D)** Abdominal CT scan in December 2025 shows no evidence of disease after salvage surgery.

**Figure 2:**
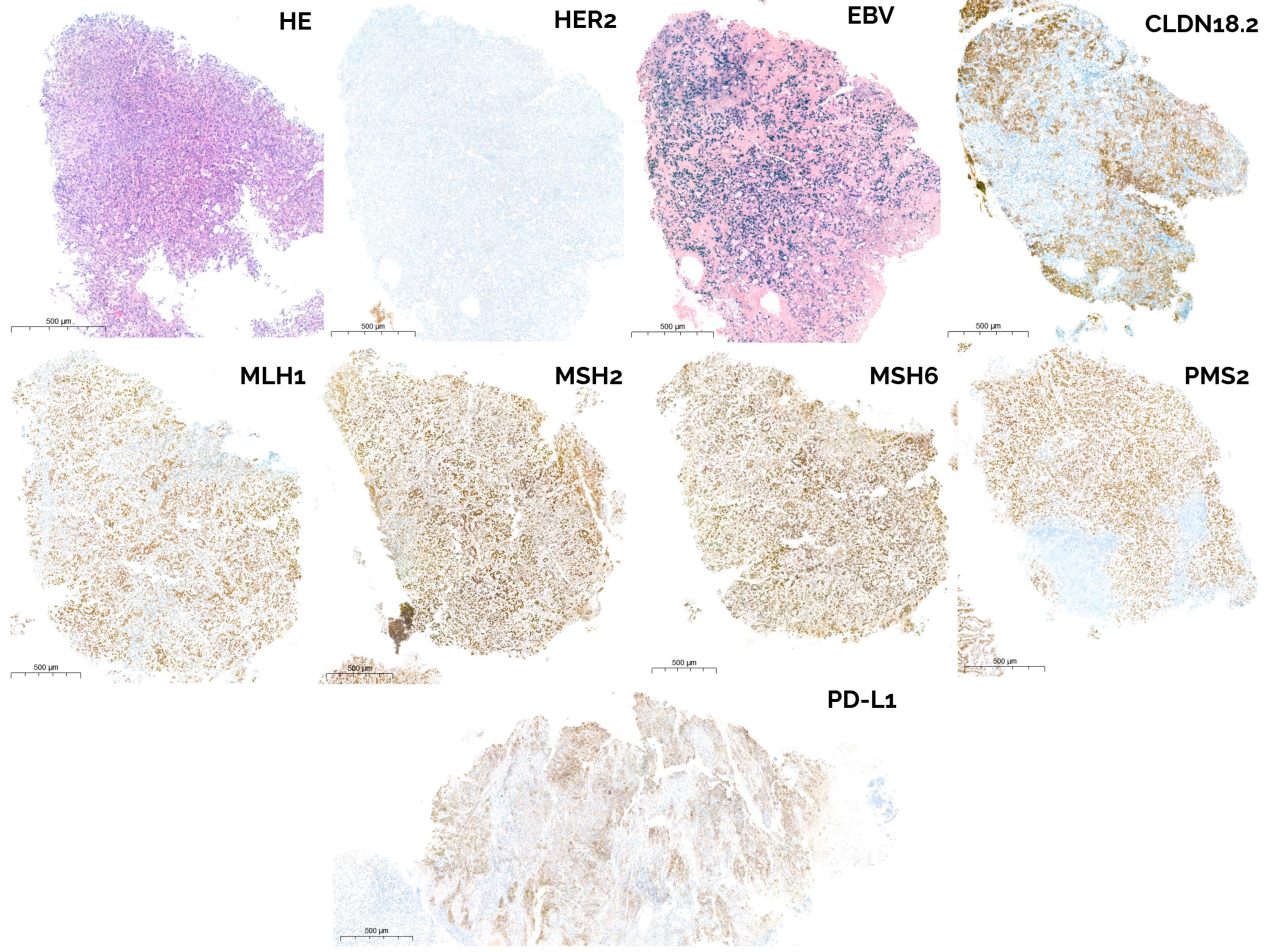
Histopathological and immunohistochemical characterization of the gastric tumor. Representative sections from the diagnostic biopsy show gastric adenocarcinoma (H&E). HER2 immunohistochemistry (IHC) shows no membranous overexpression (0+). EBER *in situ* hybridization (ISH) demonstrates strong nuclear positivity consistent with EBV-GEA. Claudin-18.2 staining reveals partial membranous expression in tumor cells (65%). MMR protein IHC (MLH1, MSH2, MSH6, PMS2) shows intact nuclear expression in tumor cells, indicating MMR proficiency (pMMR). PD-L1 IHC showed a CPS of 69. Scale bar: 500 µm.

In May 2024, following an episode of hematemesis, he was diagnosed with gastric neoplasm described on CT scan as a prominent exophytic mural lesion in the antrum, measuring 47x64x54mm (**Figure 1A**). The biopsy showed a gastric ADC with mixed histology (tubular and poorly cohesive/signet-ring cell components), pMMR, HER2-negative (0+), and EBV-positive by *in situ* hybridization (**Figure 2**). Staging with PET-CT and endoscopic ultrasound (EUS) revealed cT4aN+ resectable disease with no evidence of distant metastasis. ECOG-PS 1 at diagnosis.

After discussion at our multidisciplinary gastrointestinal tumor board, a perioperative approach with FLOT chemotherapy was initiated in June 2024, with four cycles successfully completed by August 2024. Follow-up CT scan in September 2024 demonstrated a loco-regional progression, showing a size increase from 60 mm to 77 mm. Imaging findings suggested invasion of the transverse colon and the left hepatic lobe, making the tumor unresectable (**Figure 1B**).

**Figure 3:**
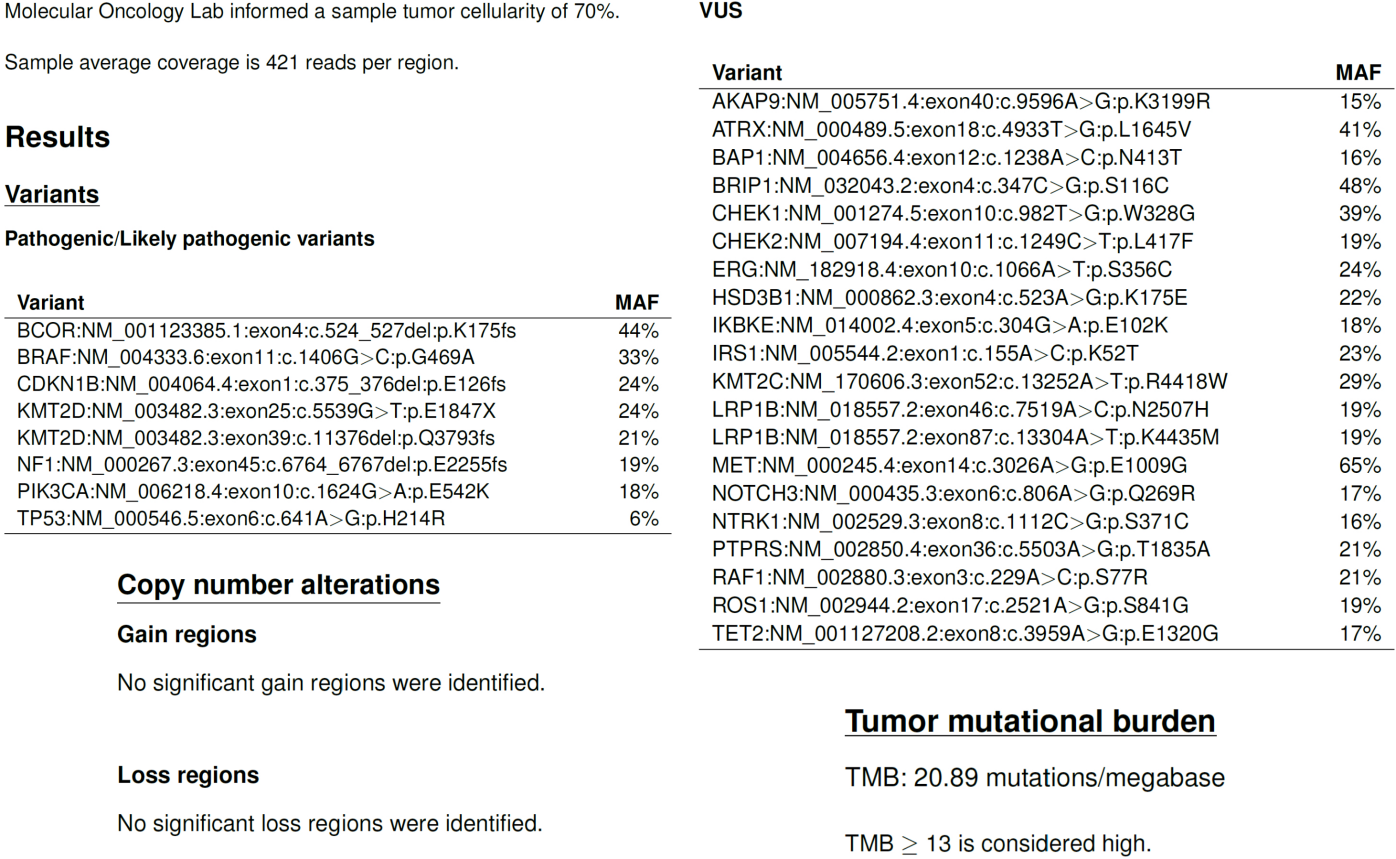
NGS profiling (in-house VHIO300 panel) of the primary gastric tumor revealed multiple somatic alterations across genes involved in chromatin regulation, cell cycle control, and oncogenic signaling pathways. Notably, an activating *PIK3CA* mutation was identified, a genomic alteration frequently reported in EBV-GEA. Variant allele frequencies (MAF) for each alteration are shown in the figure. MAF, mutant allele fraction; VUS, variant of unknown significance. Tumor mutational burden (TMB) is calculated as the number of detected and validated non-synonymous variants per megabase in the sample. Internal validation of our test established a cutoff of 13 Muts/Mb as high TMB (equivalent to 10 Muts/Mb in the FoundationOne CDx test).

Following the documented radiological progression, extended molecular profiling was performed on the initial diagnostic biopsy (**Figure 2**). This revealed a PD-L1 combined positive score (CPS) of 69, and a low CLDN18.2 expression (65% of tumor cells; the validated cutoff for zolbetuximab eligibility is ≥75% of tumor cells showing a moderate to strong 2+ or 3+ staining intensity). In-house 435-gene next-generation sequencing (NGS) panel (VHIO300) (15) was performed with no relevant findings, except for a high tumor mutational burden (TMB) of 20.89 mutations/megabase (TMB ≥ 13 considered high). Additionally, no copy number alterations were detected (**Figure 3**).

After confirmation of unresectable disease, first-line systemic therapy for advanced setting with FOLFOX plus flat-dose nivolumab (240mg iv every two weeks) was initiated. Following the third cycle, the patient developed a hypersensitivity reaction to oxaliplatin; therefore, from third cycle onwards, nivolumab was continued as monotherapy, completing 10 cycles by the end of January 2025. After the first four cycles of nivolumab, radiological assessment revealed grade 1 immune-mediated pneumonitis (CTCAE v5.0), which did not require specific management or treatment discontinuation.

After 7 cycles of nivolumab-based treatment, a CT scan performed in December 2024 demonstrated a partial response according to RECIST v1.1 criteria (-51%) (**Figure 1C**), with a tumor size decreasing to approximately 38x24 mm. The case was re-evaluated at the multidisciplinary tumor board, where the patient was considered an appropriate candidate for salvage surgery. On February 2025, he underwent total gastrectomy, omentectomy plus D2 lymphadenectomy with Roux-en-Y reconstruction.

Histopathological examination revealed a fibrotic tumor bed without residual viable tumor cells, consistent with complete pathological response (ypT0 ypN0, 0/38 lymph nodes) and tumor regression grade 1A according to the Becker classification. The former tumor site showed treatment-related changes characterized by fibrosis and inflammatory infiltrates, with no evidence of lymphovascular or perineural invasion. Additional findings included non-necrotizing granulomas in lymph nodes from stations 3, 4, 7, and 9. Histochemical stains for microorganisms were negative, and these changes were interpreted as granulomatous lymphadenitis potentially related to immunotherapy.

Postoperative complications included an esophagojejunal anastomotic leak associated with pneumoperitoneum, which required endoscopic placement of a metallic esophageal stent. Later, the patient developed septic shock secondary to acute cholecystitis (Tokyo III) requiring cholecystostomy resulting in gradual clinical recovery. After being discharged from hospital, he started follow-up with CT scans. At this point, no additional oncologic treatment was administered.

**Figure 4:**
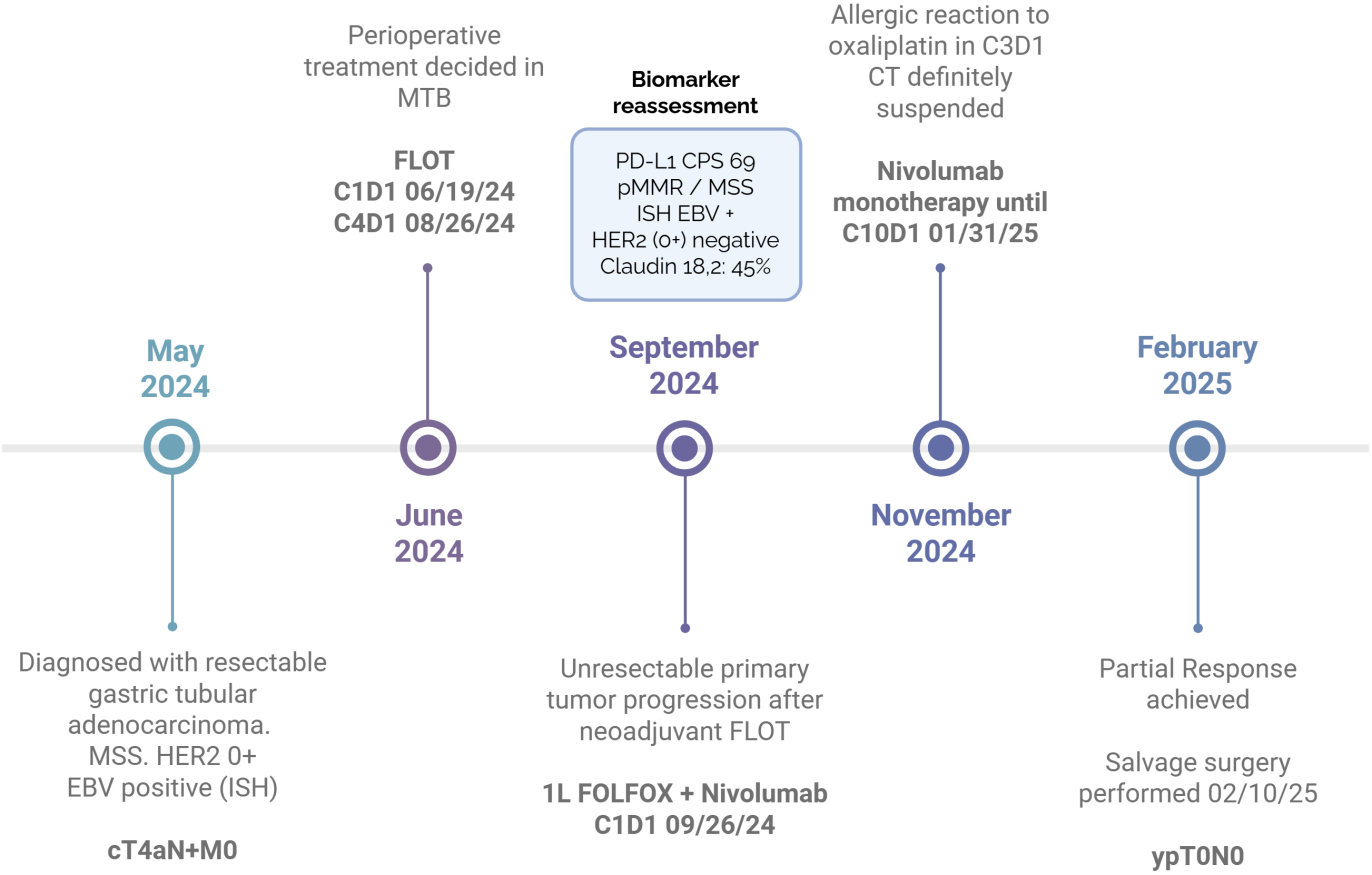
Summary timeline of case presentation. CT, chemotherapy; EBV, Epstein-Barr Virus; HER2, Human Epidermal Growth Factor Receptor 2; ISH, *In Situ* Hybridization; pMMR, Mismatch Repair proficient; MSS, Microsatellite Stability; MTB, Multidisciplinary Tumor Board; PD-L1, Programmed-death ligand 1.

The most recent CT scan, performed in December 2025, showed no evidence of disease (**Figure 1D**), and serum tumor markers remained within normal. The patient continues to improve clinically, with progressive recovery of physical activity and weight gain (**Figure 4**).

## Discussion

We report a clinical case of locally advanced gastric ADC who experienced disease progression during perioperative FLOT chemotherapy but subsequently achieved a complete pathological response after treatment with nivolumab. This outcome highlights an exceptional sensitivity to immunotherapy in a patient with microsatellite stability (MSS) tumor, likely driven by the co-occurrence of EBV positivity, high PD-L1 expression, and increased TMB.

GEA represents a heterogeneous disease characterized by an increasing number of actionable biomarkers and therapeutic options. The introduction of ICI has led to a paradigm shift, particularly in subsets of patients with PD-L1 expression and deficient MMR/MSI-high status (3).

Recent data from prospective genomic profiling studies have also spotlighted Epstein-Barr virus (EBV) as a potential predictive biomarker of ICI efficacy (14, 16). In a cohort of patients with advanced gastric cancer treated with immune checkpoint inhibitors, EBV positivity in pMMR tumors was associated with significantly improved response rates and survival outcomes compared with EBV-negative pMMR tumors, suggesting that EBV infection may identify a subgroup of MSS gastric cancers particularly sensitive to immunotherapy (17).

EBV-GEA often displays a distinct tumor microenvironment characterized by increased immune infiltration and frequent PD-L1 expression, likely driven by viral antigen presentation and inflammatory signaling pathways (18, 19). Interestingly, durable responses to immunotherapy have been described even in EBV-positive tumors with relatively low TMB, suggesting that viral-driven oncogenesis itself may enhance tumor immunogenicity (20). Nevertheless, EBV-GEA is not biologically uniform. The immune landscape may vary according to histological subtype, with lymphoepithelioma-like carcinomas typically exhibiting abundant tumor-infiltrating lymphocytes and higher PD-L1 expression, whereas intestinal or poorly cohesive ADC tend to be less immunogenic (18, 19). This heterogeneity may partly explain the variability in clinical responses to ICI observed in EBV-GEA.

In HER2-negative and PD-L1 positive advanced GEA, standard regimens include fluoropyrimidine-platinum-based chemotherapy combined with immunotherapy (nivolumab, pembrolizumab or tislelizumab); guided by CPS and TAP score thresholds (21).

In the exploratory analysis of the phase III CheckMate 649 trial, although the limited sample size, patients with hypermutated tumors (n=31) derived the greatest OS benefit from chemoimmunotherapy (HR 0.37; 95% CI, 0.15-0.90) followed by EBV-positive tumors (n=34), which also showed a trend toward benefit from the addition of nivolumab to chemotherapy (HR 0.61; 95% CI, 0.26-1.45) [8.22]. In our case, the achievement of a complete pathological response in an EBV-positive tumor reinforces the role of EBV as a potential marker of immunotherapy sensitivity. Despite the limitations of PD-L1 CPS as a predictive biomarker for ICI efficacy, a recent meta-analysis of pivotal phase III trials reported a strong benefit in patients with PD-L1 CPS≥10 (10).

Along with that, TMB-high tumors (n=57) demonstrated improved outcomes with a HR 0.48 (95% CI, 0.25-0.91), compared to TMB-low patients (HR 0.85; 95% CI, 0.72-1.00). Importantly, the effect of TMB remained even after excluding MSI-high tumors, with TMB-high/MSS cases (n=26) achieving a HR of 0.57 (95% CI, 0.21-1.51), although the subgroup population is small (22). Our patient was TMB-high (20.89 mut/MB), MSS and PD-L1 high, a combination associated with an increased likelihood of response as shown in this analysis. The profound pathological remission observed suggests that TMB, even in MSS tumors, may serve as a relevant predictor of immunotherapy benefit.

In MSS tumors, chemoimmunotherapy achieved a modest but statistically significant survival benefit over chemotherapy alone (mOS 13.8 vs 11.5 months; HR 0.79, 95% CI 0.71-0.89), which was clearly inferior to the benefit achieved in MSI-high tumors (mOS 38.7 vs 12.3 months; HR 0.34, 95% CI 016-0-74) (22).

In our case, a deep response was achieved by the end of the seventh cycle; however, treatment was continued for three additional cycles, raising the question: is there enough evidence on optimal duration of ‘neoadjuvant’ immunotherapy-based treatment to achieve the deepest benefit?

In the literature, neoadjuvant immunotherapy approach in deficient MMR tumors (including gastroesophageal malignancies) has shown a positive correlation between longer anti-PD-1/PD-L1 exposure and complete-response rates (23, 24). In deficient MMR/MSI-high colorectal cancer, pooled data from neoadjuvant immunotherapy studies show a positive correlation between treatment duration and response, with complete response rates of approximately 80% after more than four months of therapy, increasing to nearly 100% when treatment is extended to around seven months (23, 25). These findings support response-adapted strategies and highlight the need to address this question prospectively in EBV-GEA.

Another open question is whether chemotherapy should be combined with PD-1 inhibitors or whether PD-1 blockade alone is sufficient. In MSI-high/deficient MMR tumors, the optimal approach remains uncertain; and chemoimmunotherapy may be recommended in the presence of high-symptom burden or involvement of vital organs (26). However, in EBV-GEA, this question remains unanswered.

Several ongoing studies are addressing whether PD-1 blockade should be combined with chemotherapy in EBV-positive gastric cancer. A recent single-center study reported that first-line nivolumab plus chemotherapy in EBV-positive advanced gastric cancer showed promising efficacy, suggesting that EBV status may predict response to chemoimmunotherapy (27). Additionally, preliminary data from ongoing trials are exploring preoperative or adjuvant PD-1 inhibitors in biomarker-selected populations, including EBV-positive, PD-L1 high, or MSI-high gastric tumors (28, 29), aiming to define whether ICI alone or in combination with chemotherapy is optimal. These studies may help clarify treatment strategies specifically for EBV-GEA.

Despite the implementation of perioperative FLOT chemotherapy as the standard of care, the 5-year OS remains below 50%, underscoring the substantial unmet clinical need in this population. Improved molecular characterization and biomarker-driven treatment strategies may help refine patient selection and enhance outcomes (30, 31).

Although EBV status is not a validated predictive biomarker in gastric cancer, EBV-positive tumors showed a trend toward OS benefit with chemoimmunotherapy. EBV testing could identify a subgroup population with potential higher sensitivity to immunotherapy. Our case also illustrates how the co-occurrence of EBV positivity, TMB-high status, and high PD-L1 expression may define a particularly immunoresponsive subgroup among pMMR/MSS GEA.

In summary, this favorable biomarker combination likely contributed to the pathological response observed in a patient with an otherwise poor theoretical prognosis after progression on preoperative triplet chemotherapy. This reinforces the importance of comprehensive biomarker assessment and supports further investigation of immunotherapy strategies in EBV-GEA.

## Data Availability

The datasets presented in this article are not readily available because of ethical and privacy restrictions. Requests to access the datasets should be directed to the corresponding author/s.
